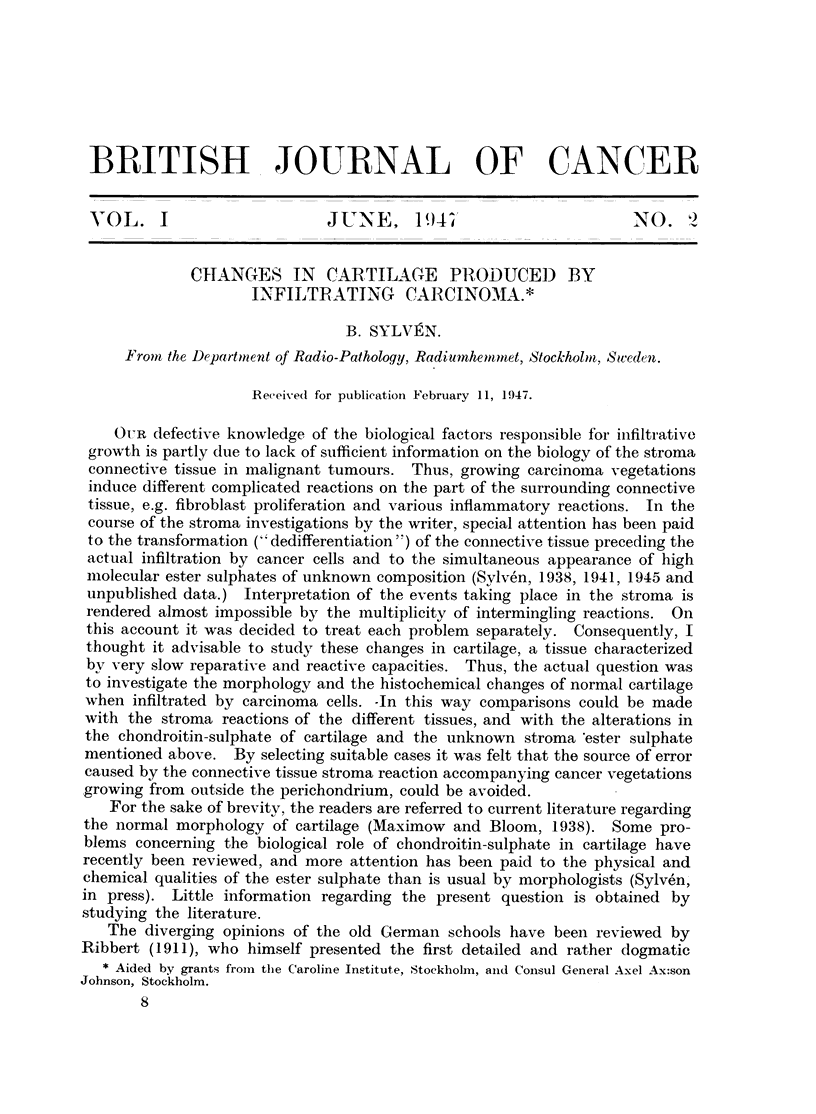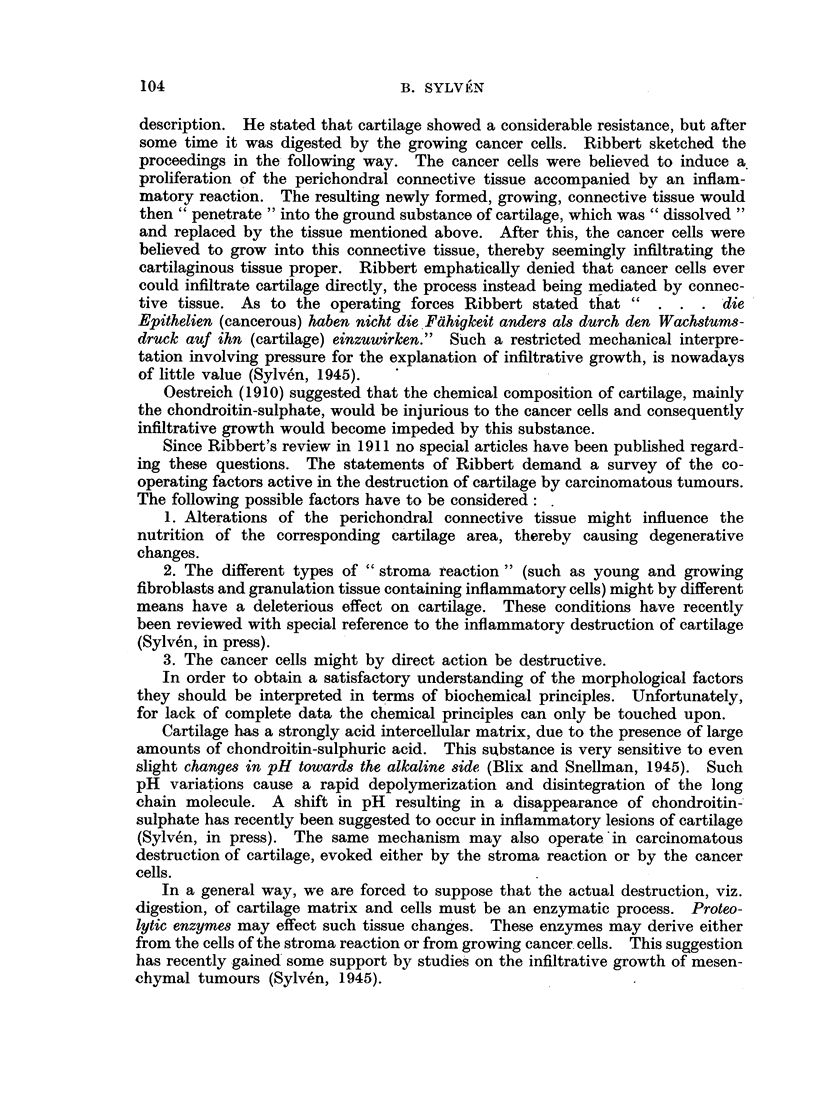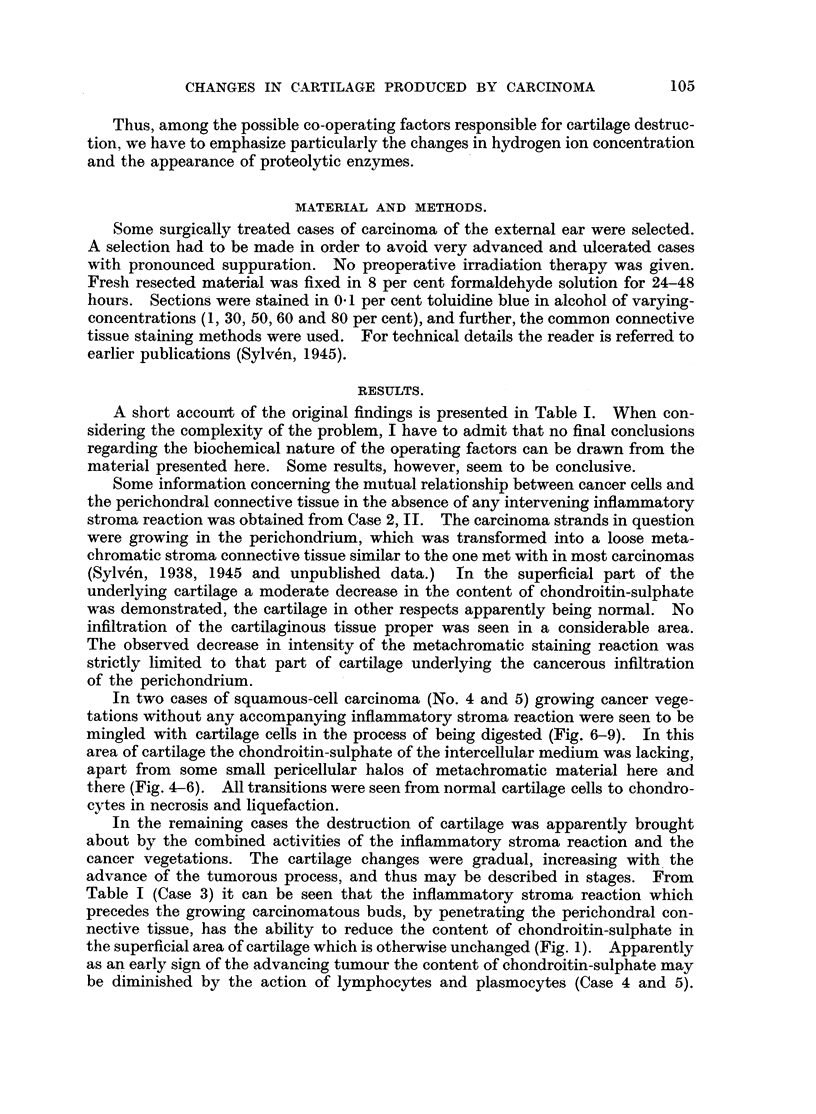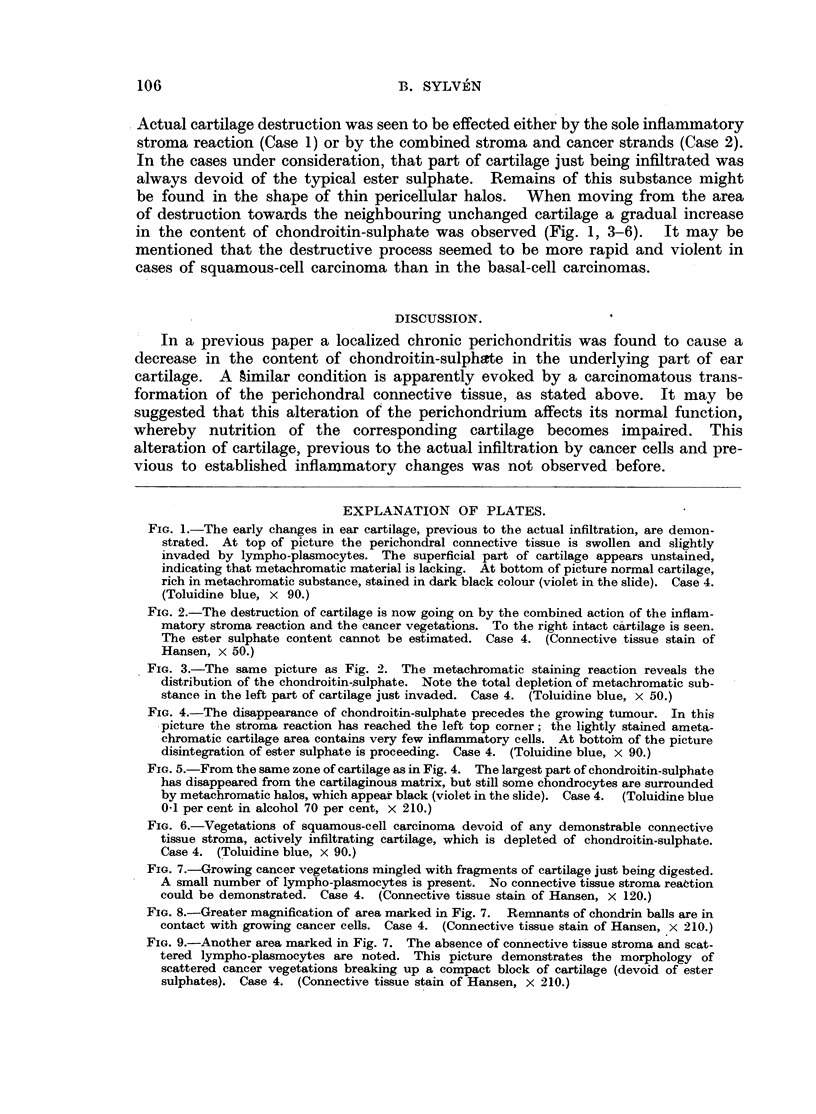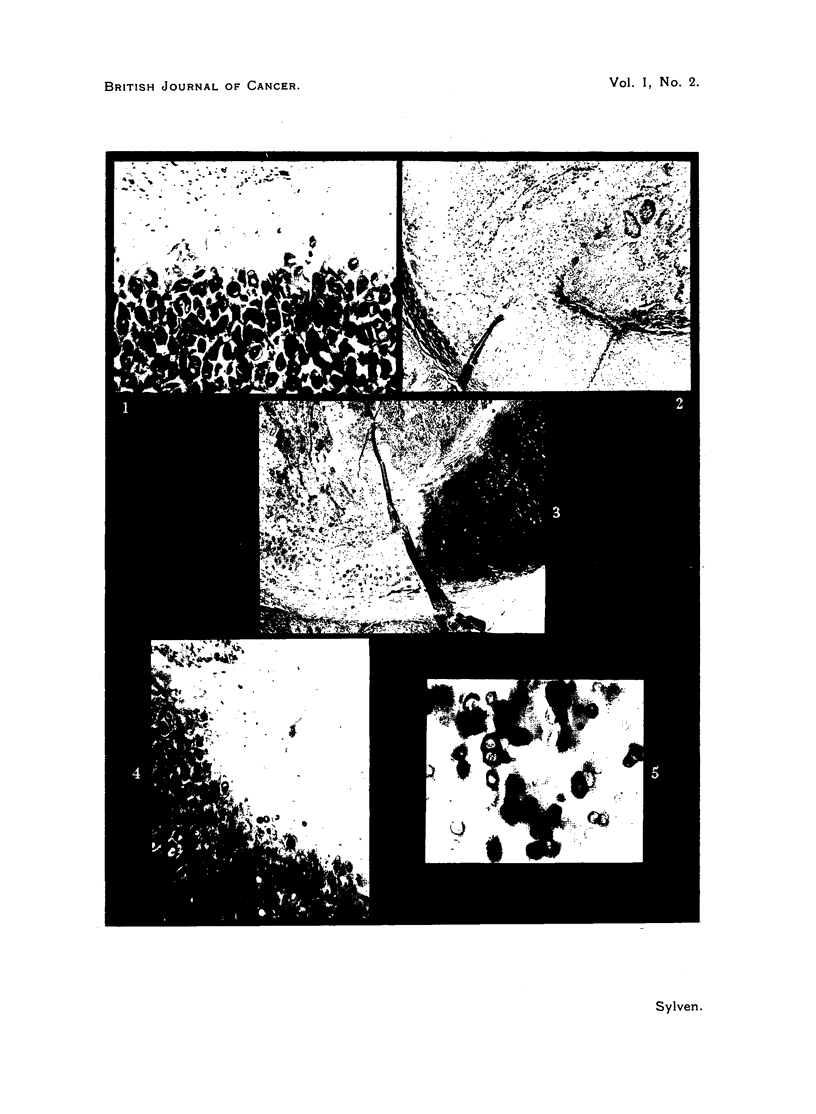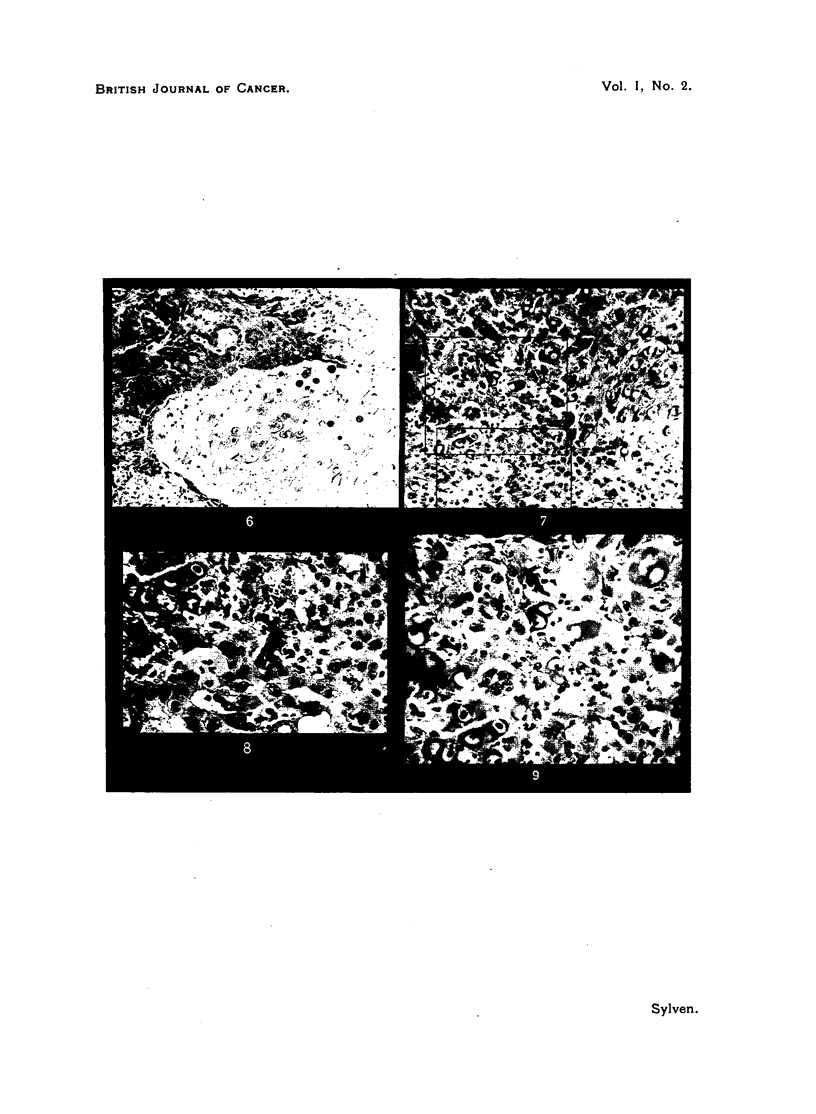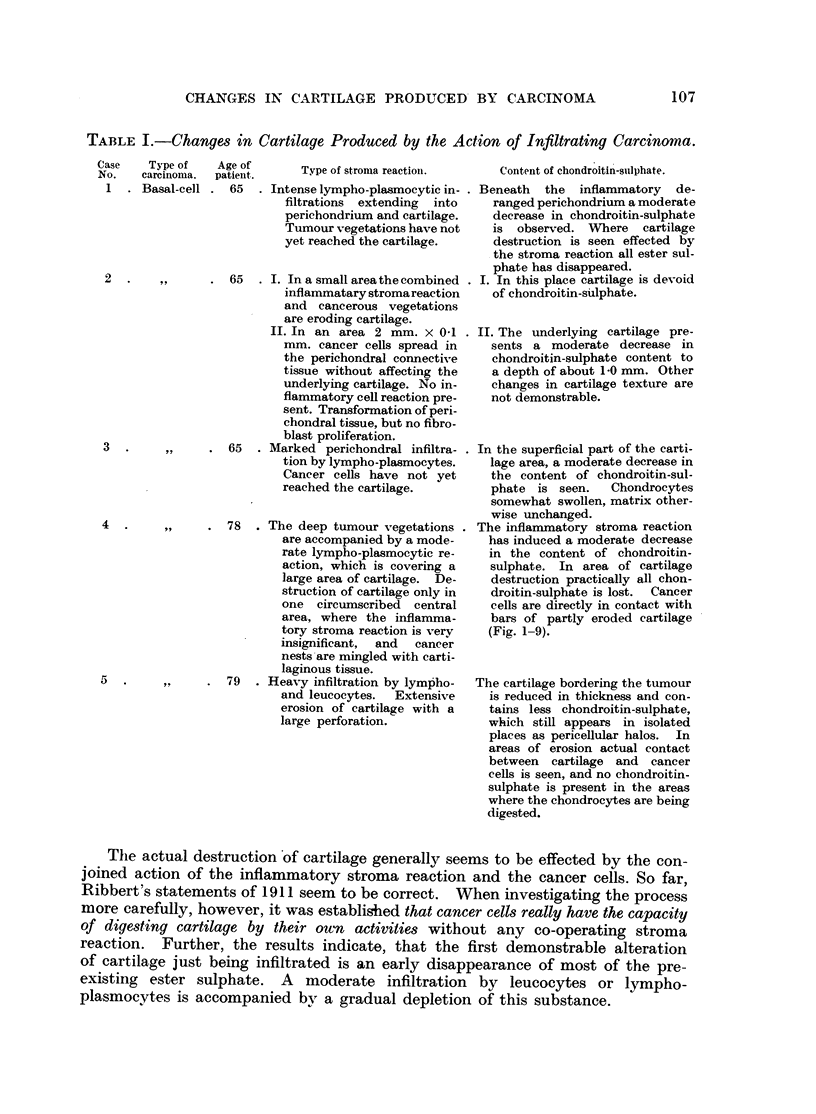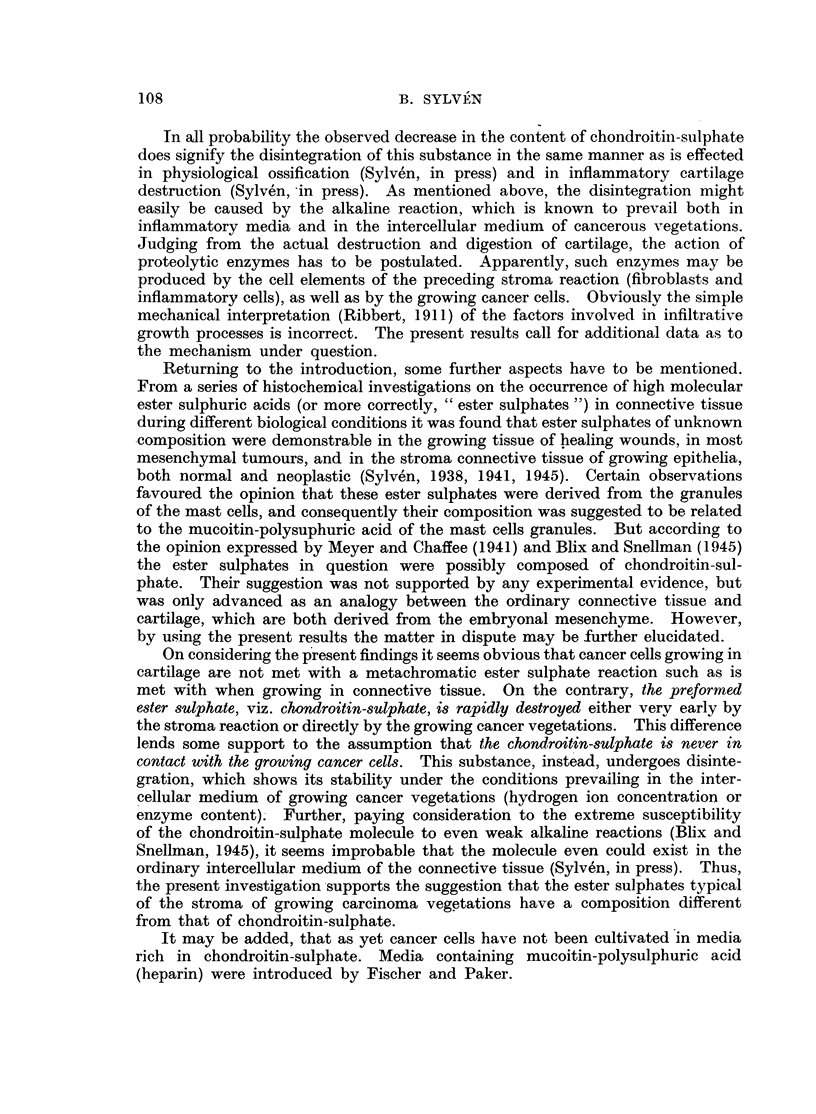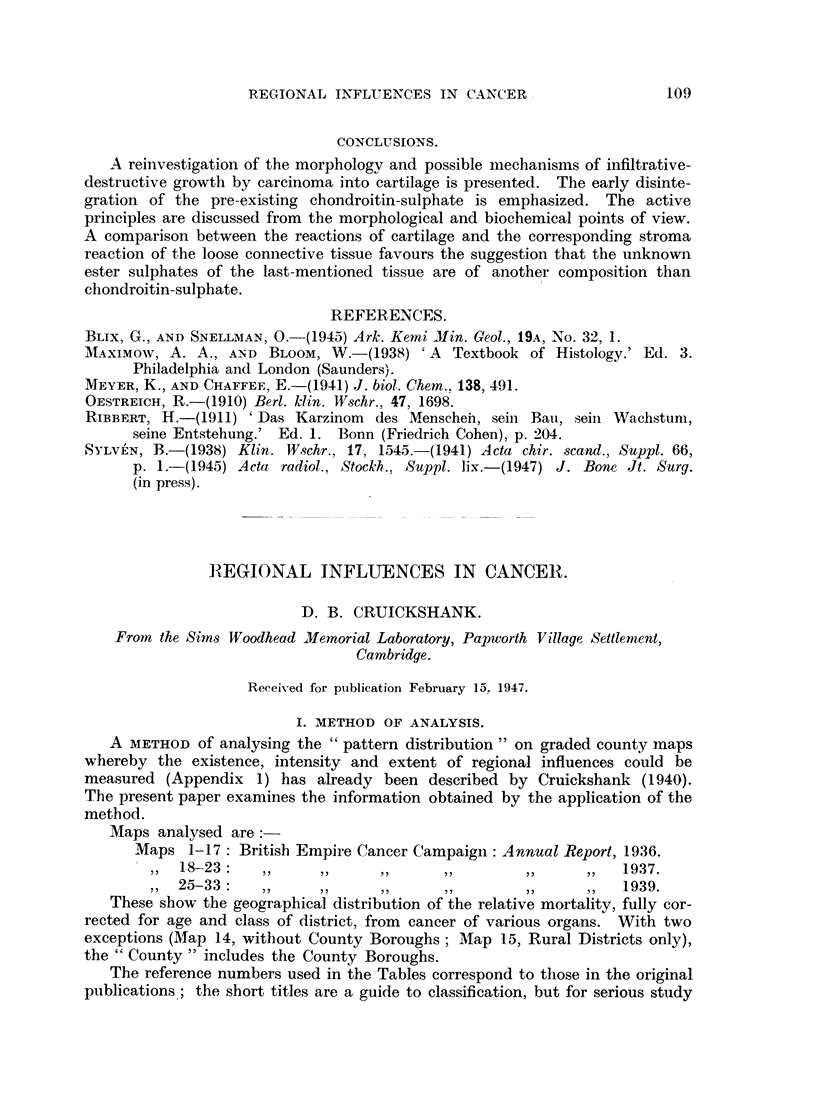# Changes in Cartilage Produced by Infiltrating Carcinoma*

**DOI:** 10.1038/bjc.1947.13

**Published:** 1947-06

**Authors:** B. Sylvén

## Abstract

**Images:**


// BRITISH JOURNAL OF CANCER

VOL. I JUNE, 1947 NO. 2

### CHANGES IN CARTILAGE PRODUCED BY INFILTRATING CARCINOMA.*

B. SYLVÉN.

*From the Department of Radio-Pathology, Radiumhemmet, Stockholm, Sweden.*

Received for publication February 11, 1947.

OUR defective knowledge of the biological factors responsible for infiltrative growth is partly due to lack of sufficient information on the biology of the stroma connective tissue in malignant tumours. Thus, growing carcinoma vegetations induce different complicated reactions on the part of the surrounding connective tissue, e.g. fibroblast proliferation and various inflammatory reactions. In the course of the stroma investigations by the writer, special attention has been paid to the transformation ("dedifferentiation") of the connective tissue preceding the actual infiltration by cancer cells and to the simultaneous appearance of high molecular ester sulphates of unknown composition (Sylvén, 1938, 1941, 1945 and unpublished data.) Interpretation of the events taking place in the stroma is rendered almost impossible by the multiplicity of intermingling reactions. On this account it was decided to treat each problem separately. Consequently, I thought it advisable to study these changes in cartilage, a tissue characterized by very slow reparative and reactive capacities. Thus, the actual question was to investigate the morphology and the histochemical changes of normal cartilage when infiltrated by carcinoma cells. In this way comparisons could be made with the stroma reactions of the different tissues, and with the alterations in the chondroitin-sulphate of cartilage and the unknown stroma ester sulphate mentioned above. By selecting suitable cases it was felt that the source of error caused by the connective tissue stroma reaction accompanying cancer vegetations growing from outside the perichondrium, could be avoided.

For the sake of brevity, the readers are referred to current literature regarding the normal morphology of cartilage (Maximow and Bloom, 1938). Some problems concerning the biological role of chondroitin-sulphate in cartilage have recently been reviewed, and more attention has been paid to the physical and chemical qualities of the ester sulphate than is usual by morphologists (Sylvén, in press). Little information regarding the present question is obtained by studying the literature.

The diverging opinions of the old German schools have been reviewed by Ribbert (1911), who himself presented the first detailed and rather dogmatic

* Aided by grants from the Caroline Institute, Stockholm, and Consul General Axel Ax:son Johnson, Stockholm.

description. He stated that cartilage showed a considerable resistance, but after some time it was digested by the growing cancer cells. Ribbert sketched the proceedings in the following way. The cancer cells were believed to induce a proliferation of the perichondral connective tissue accompanied by an inflammatory reaction. The resulting newly formed, growing, connective tissue would then " penetrate " into the ground substance of cartilage, which was " dissolved " and replaced by the tissue mentioned above. After this, the cancer cells were believed to grow into this connective tissue, thereby seemingly infiltrating the cartilaginous tissue proper. Ribbert emphatically denied that cancer cells ever could infiltrate cartilage directly, the process instead being mediated by connective tissue. As to the operating forces Ribbert stated that " . . . *die Epithelien* (cancerous) *haben nicht die Fähigkeit anders als durch den Wachstumsdruck auf ihn* (cartilage) *einzuwirken.*" Such a restricted mechanical interpretation involving pressure for the explanation of infiltrative growth, is nowadays of little value (Sylvén, 1945).

Oestreich (1910) suggested that the chemical composition of cartilage, mainly the chondroitin-sulphate, would be injurious to the cancer cells and consequently infiltrative growth would become impeded by this substance.

Since Ribbert's review in 1911 no special articles have been published regarding these questions. The statements of Ribbert demand a survey of the cooperating factors active in the destruction of cartilage by carcinomatous tumours. The following possible factors have to be considered :

1. Alterations of the perichondral connective tissue might influence the nutrition of the corresponding cartilage area, thereby causing degenerative changes.

2. The different types of " stroma reaction " (such as young and growing fibroblasts and granulation tissue containing inflammatory cells) might by different means have a deleterious effect on cartilage. These conditions have recently been reviewed with special reference to the inflammatory destruction of cartilage (Sylvén, in press).

3. The cancer cells might by direct action be destructive.

In order to obtain a satisfactory understanding of the morphological factors they should be interpreted in terms of biochemical principles. Unfortunately, for lack of complete data the chemical principles can only be touched upon.

Cartilage has a strongly acid intercellular matrix, due to the presence of large amounts of chondroitin-sulphuric acid. This substance is very sensitive to even slight *changes in pH towards the alkaline side* (Blix and Snellman, 1945). Such pH variations cause a rapid depolymerization and disintegration of the long chain molecule. A shift in pH resulting in a disappearance of chondroitin-sulphate has recently been suggested to occur in inflammatory lesions of cartilage (Sylvén, in press). The same mechanism may also operate in carcinomatous destruction of cartilage, evoked either by the stroma reaction or by the cancer cells.

In a general way, we are forced to suppose that the actual destruction, viz. digestion, of cartilage matrix and cells must be an enzymatic process. *Proteolytic enzymes* may effect such tissue changes. These enzymes may derive either from the cells of the stroma reaction or from growing cancer cells. This suggestion has recently gained some support by studies on the infiltrative growth of mesenchymal tumours (Sylvén, 1945).

Thus, among the possible co-operating factors responsible for cartilage destruction, we have to emphasize particularly the changes in hydrogen ion concentration and the appearance of proteolytic enzymes.

### MATERIAL AND METHODS.

Some surgically treated cases of carcinoma of the external ear were selected. A selection had to be made in order to avoid very advanced and ulcerated cases with pronounced suppuration. No preoperative irradiation therapy was given. Fresh resected material was fixed in 8 per cent formaldehyde solution for 24–48 hours. Sections were stained in 0·1 per cent toluidine blue in alcohol of varying-concentrations (1, 30, 50, 60 and 80 per cent), and further, the common connective tissue staining methods were used. For technical details the reader is referred to earlier publications (Sylvén, 1945).

### RESULTS.

A short account of the original findings is presented in Table I. When considering the complexity of the problem, I have to admit that no final conclusions regarding the biochemical nature of the operating factors can be drawn from the material presented here. Some results, however, seem to be conclusive.

Some information concerning the mutual relationship between cancer cells and the perichondral connective tissue in the absence of any intervening inflammatory stroma reaction was obtained from Case 2, II. The carcinoma strands in question were growing in the perichondrium, which was transformed into a loose metachromatic stroma connective tissue similar to the one met with in most carcinomas (Sylvén, 1938, 1945 and unpublished data.) In the superficial part of the underlying cartilage a moderate decrease in the content of chondroitin-sulphate was demonstrated, the cartilage in other respects apparently being normal. No infiltration of the cartilaginous tissue proper was seen in a considerable area. The observed decrease in intensity of the metachromatic staining reaction was strictly limited to that part of cartilage underlying the cancerous infiltration of the perichondrium.

In two cases of squamous-cell carcinoma (No. 4 and 5) growing cancer vegetations without any accompanying inflammatory stroma reaction were seen to be mingled with cartilage cells in the process of being digested (Fig. 6–9). In this area of cartilage the chondroitin-sulphate of the intercellular medium was lacking, apart from some small pericellular halos of metachromatic material here and there (Fig. 4–6). All transitions were seen from normal cartilage cells to chondrocytes in necrosis and liquefaction.

In the remaining cases the destruction of cartilage was apparently brought about by the combined activities of the inflammatory stroma reaction and the cancer vegetations. The cartilage changes were gradual, increasing with the advance of the tumorous process, and thus may be described in stages. From Table I (Case 3) it can be seen that the inflammatory stroma reaction which precedes the growing carcinomatous buds, by penetrating the perichondral connective tissue, has the ability to reduce the content of chondroitin-sulphate in the superficial area of cartilage which is otherwise unchanged (Fig. 1). Apparently as an early sign of the advancing tumour the content of chondroitin-sulphate may be diminished by the action of lymphocytes and plasmocytes (Case 4 and 5).

Actual cartilage destruction was seen to be effected either by the sole inflammatory stroma reaction (Case 1) or by the combined stroma and cancer strands (Case 2). In the cases under consideration, that part of cartilage just being infiltrated was always devoid of the typical ester sulphate. Remains of this substance might be found in the shape of thin pericellular halos. When moving from the area of destruction towards the neighbouring unchanged cartilage a gradual increase in the content of chondroitin-sulphate was observed (Fig. 1, 3–6). It may be mentioned that the destructive process seemed to be more rapid and violent in cases of squamous-cell carcinoma than in the basal-cell carcinomas.

### DISCUSSION.

In a previous paper a localized chronic perichondritis was found to cause a decrease in the content of chondroitin-sulphate in the underlying part of ear cartilage. A similar condition is apparently evoked by a carcinomatous transformation of the perichondral connective tissue, as stated above. It may be suggested that this alteration of the perichondrium affects its normal function, whereby nutrition of the corresponding cartilage becomes impaired. This alteration of cartilage, previous to the actual infiltration by cancer cells and previous to established inflammatory changes was not observed before.

---

### EXPLANATION OF PLATES.

FIG. 1.—The early changes in ear cartilage, previous to the actual infiltration, are demonstrated. At top of picture the perichondral connective tissue is swollen and slightly invaded by lympho-plasmocytes. The superficial part of cartilage appears unstained, indicating that metachromatic material is lacking. At bottom of picture normal cartilage, rich in metachromatic substance, stained in dark black colour (violet in the slide). Case 4. (Toluidine blue, × 90.)

FIG. 2.—The destruction of cartilage is now going on by the combined action of the inflammatory stroma reaction and the cancer vegetations. To the right intact cartilage is seen. The ester sulphate content cannot be estimated. Case 4. (Connective tissue stain of Hansen, × 50.)

FIG. 3.—The same picture as Fig. 2. The metachromatic staining reaction reveals the distribution of the chondroitin-sulphate. Note the total depletion of metachromatic substance in the left part of cartilage just invaded. Case 4. (Toluidine blue, × 50.)

FIG. 4.—The disappearance of chondroitin-sulphate precedes the growing tumour. In this picture the stroma reaction has reached the left top corner; the lightly stained ametachromatic cartilage area contains very few inflammatory cells. At bottom of the picture disintegration of ester sulphate is proceeding. Case 4. (Toluidine blue, × 90.)

FIG. 5.—From the same zone of cartilage as in Fig. 4. The largest part of chondroitin-sulphate has disappeared from the cartilaginous matrix, but still some chondrocytes are surrounded by metachromatic halos, which appear black (violet in the slide). Case 4. (Toluidine blue 0·1 per cent in alcohol 70 per cent, × 210.)

FIG. 6.—Vegetations of squamous-cell carcinoma devoid of any demonstrable connective tissue stroma, actively infiltrating cartilage, which is depleted of chondroitin-sulphate. Case 4. (Toluidine blue, × 90.)

FIG. 7.—Growing cancer vegetations mingled with fragments of cartilage just being digested. A small number of lympho-plasmocytes is present. No connective tissue stroma reaction could be demonstrated. Case 4. (Connective tissue stain of Hansen, × 120.)

FIG. 8.—Greater magnification of area marked in Fig. 7. Remnants of chondrin balls are in contact with growing cancer cells. Case 4. (Connective tissue stain of Hansen, × 210.)

FIG. 9.—Another area marked in Fig. 7. The absence of connective tissue stroma and scattered lympho-plasmocytes are noted. This picture demonstrates the morphology of scattered cancer vegetations breaking up a compact block of cartilage (devoid of ester sulphates). Case 4. (Connective tissue stain of Hansen, × 210.)

Sylven.

6

7

8

9

TABLE I.—*Changes in Cartilage Produced by the Action of Infiltrating Carcinoma.*

| Case No. | Type of carcinoma. | Age of patient. | Type of stroma reaction. | Content of chondroitin-sulphate. |
|---|---|---|---|---|
| 1 | Basal-cell | 65 | Intense lympho-plasmocytic infiltrations extending into perichondrium and cartilage. Tumour vegetations have not yet reached the cartilage. | Beneath the inflammatory deranged perichondrium a moderate decrease in chondroitin-sulphate is observed. Where cartilage destruction is seen effected by the stroma reaction all ester sulphate has disappeared. |
| 2 | ,, | 65 | I. In a small area the combined inflammatary stroma reaction and cancerous vegetations are eroding cartilage. | I. In this place cartilage is devoid of chondroitin-sulphate. |
| | | | II. In an area 2 mm. × 0·1 mm. cancer cells spread in the perichondral connective tissue without affecting the underlying cartilage. No inflammatory cell reaction present. Transformation of perichondral tissue, but no fibroblast proliferation. | II. The underlying cartilage presents a moderate decrease in chondroitin-sulphate content to a depth of about 1·0 mm. Other changes in cartilage texture are not demonstrable. |
| 3 | ,, | 65 | Marked perichondral infiltration by lympho-plasmocytes. Cancer cells have not yet reached the cartilage. | In the superficial part of the cartilage area, a moderate decrease in the content of chondroitin-sulphate is seen. Chondrocytes somewhat swollen, matrix otherwise unchanged. |
| 4 | ,, | 78 | The deep tumour vegetations are accompanied by a moderate lympho-plasmocytic reaction, which is covering a large area of cartilage. Destruction of cartilage only in one circumscribed central area, where the inflammatory stroma reaction is very insignificant, and cancer nests are mingled with cartilaginous tissue. | The inflammatory stroma reaction has induced a moderate decrease in the content of chondroitin-sulphate. In area of cartilage destruction practically all chondroitin-sulphate is lost. Cancer cells are directly in contact with bars of partly eroded cartilage (Fig. 1–9). |
| 5 | ,, | 79 | Heavy infiltration by lympho- and leucocytes. Extensive erosion of cartilage with a large perforation. | The cartilage bordering the tumour is reduced in thickness and contains less chondroitin-sulphate, which still appears in isolated places as pericellular halos. In areas of erosion actual contact between cartilage and cancer cells is seen, and no chondroitin-sulphate is present in the areas where the chondrocytes are being digested. |

The actual destruction of cartilage generally seems to be effected by the conjoined action of the inflammatory stroma reaction and the cancer cells. So far, Ribbert's statements of 1911 seem to be correct. When investigating the process more carefully, however, it was established *that cancer cells really have the capacity of digesting cartilage by their own activities* without any co-operating stroma reaction. Further, the results indicate, that the first demonstrable alteration of cartilage just being infiltrated is an early disappearance of most of the pre-existing ester sulphate. A moderate infiltration by leucocytes or lympho-plasmocytes is accompanied by a gradual depletion of this substance.

In all probability the observed decrease in the content of chondroitin-sulphate does signify the disintegration of this substance in the same manner as is effected in physiological ossification (Sylvén, in press) and in inflammatory cartilage destruction (Sylvén, in press). As mentioned above, the disintegration might easily be caused by the alkaline reaction, which is known to prevail both in inflammatory media and in the intercellular medium of cancerous vegetations. Judging from the actual destruction and digestion of cartilage, the action of proteolytic enzymes has to be postulated. Apparently, such enzymes may be produced by the cell elements of the preceding stroma reaction (fibroblasts and inflammatory cells), as well as by the growing cancer cells. Obviously the simple mechanical interpretation (Ribbert, 1911) of the factors involved in infiltrative growth processes is incorrect. The present results call for additional data as to the mechanism under question.

Returning to the introduction, some further aspects have to be mentioned. From a series of histochemical investigations on the occurrence of high molecular ester sulphuric acids (or more correctly, " ester sulphates ") in connective tissue during different biological conditions it was found that ester sulphates of unknown composition were demonstrable in the growing tissue of healing wounds, in most mesenchymal tumours, and in the stroma connective tissue of growing epithelia, both normal and neoplastic (Sylvén, 1938, 1941, 1945). Certain observations favoured the opinion that these ester sulphates were derived from the granules of the mast cells, and consequently their composition was suggested to be related to the mucoitin-polysuphuric acid of the mast cells granules. But according to the opinion expressed by Meyer and Chaffee (1941) and Blix and Snellman (1945) the ester sulphates in question were possibly composed of chondroitin-sulphate. Their suggestion was not supported by any experimental evidence, but was only advanced as an analogy between the ordinary connective tissue and cartilage, which are both derived from the embryonal mesenchyme. However, by using the present results the matter in dispute may be further elucidated.

On considering the present findings it seems obvious that cancer cells growing in cartilage are not met with a metachromatic ester sulphate reaction such as is met with when growing in connective tissue. On the contrary, *the preformed ester sulphate*, viz. *chondroitin-sulphate, is rapidly destroyed* either very early by the stroma reaction or directly by the growing cancer vegetations. This difference lends some support to the assumption that *the chondroitin-sulphate is never in contact with the growing cancer cells*. This substance, instead, undergoes disintegration, which shows its stability under the conditions prevailing in the intercellular medium of growing cancer vegetations (hydrogen ion concentration or enzyme content). Further, paying consideration to the extreme susceptibility of the chondroitin-sulphate molecule to even weak alkaline reactions (Blix and Snellman, 1945), it seems improbable that the molecule even could exist in the ordinary intercellular medium of the connective tissue (Sylvén, in press). Thus, the present investigation supports the suggestion that the ester sulphates typical of the stroma of growing carcinoma vegetations have a composition different from that of chondroitin-sulphate.

It may be added, that as yet cancer cells have not been cultivated in media rich in chondroitin-sulphate. Media containing mucoitin-polysulphuric acid (heparin) were introduced by Fischer and Paker.

### CONCLUSIONS.

A reinvestigation of the morphology and possible mechanisms of infiltrative-destructive growth by carcinoma into cartilage is presented. The early disintegration of the pre-existing chondroitin-sulphate is emphasized. The active principles are discussed from the morphological and biochemical points of view. A comparison between the reactions of cartilage and the corresponding stroma reaction of the loose connective tissue favours the suggestion that the unknown ester sulphates of the last-mentioned tissue are of another composition than chondroitin-sulphate.

### REFERENCES.

BLIX, G., AND SNELLMAN, O.—(1945) *Ark. Kemi Min. Geol.*, **19A**, No. 32, 1.
MAXIMOW, A. A., AND BLOOM, W.—(1938) 'A Textbook of Histology.' Ed. 3. Philadelphia and London (Saunders).
MEYER, K., AND CHAFFEE, E.—(1941) *J. biol. Chem.*, **138**, 491.
OESTREICH, R.—(1910) *Berl. klin. Wschr.*, **47**, 1698.
RIBBERT, H.—(1911) 'Das Karzinom des Menschen, sein Bau, sein Wachstum, seine Entstehung.' Ed. 1. Bonn (Friedrich Cohen), p. 204.
SYLVÉN, B.—(1938) *Klin. Wschr.*, **17**, 1545.—(1941) *Acta chir. scand.*, *Suppl.* 66, p. 1.—(1945) *Acta radiol.*, *Stockh.*, *Suppl.* lix.—(1947) *J. Bone Jt. Surg.* (in press).